# The dynamics of scarlet fever in The Netherlands, 1906–1920: a historical analysis

**DOI:** 10.1098/rsos.220030

**Published:** 2022-11-02

**Authors:** Scott A. McDonald, Maarten van Wijhe, Brechje de Gier, Hester Korthals Altes, Bart J. M. Vlaminckx, Susan Hahné, Jacco Wallinga

**Affiliations:** ^1^ Centre for Infectious Disease Control, Netherlands National Institute for Public Health and the Environment, Bilthoven, The Netherlands; ^2^ Roskilde University, Roskilde, Denmark; ^3^ St. Antonius Hospital, Nieuwegein, The Netherlands; ^4^ Department of Biomedical Data Sciences, Leiden University Medical Center, Leiden, The Netherlands

**Keywords:** scarlet fever, *S. pyogenes*, transmission rate, historical analysis, The Netherlands

## Abstract

**Background.** Scarlet fever, an infectious disease caused by *Streptococcus pyogenes*, largely disappeared in developed countries during the twentieth century. In recent years, scarlet fever is on the rise again, and there is a need for a better understanding of possible factors driving transmission. **Methods.** Using historical case notification data from the three largest cities in The Netherlands (Amsterdam, Rotterdam and The Hague) from 1906 to 1920, we inferred the transmission rate for scarlet fever using time-series susceptible-infected-recovered (TSIR) methods. Through additive regression modelling, we investigated the contributions of meteorological variables and school term times to transmission rates. **Results.** Estimated transmission rates varied by city, and were highest overall for Rotterdam, the most densely populated city at that time. High temperature, seasonal precipitation levels and school term timing were associated with transmission rates, but the roles of these factors were limited and not consistent over all three cities. **Conclusions.** While weather factors alone can only explain a small portion of the variability in transmission rates, these results help understand the historical dynamics of scarlet fever infection in an era with less advanced sanitation and no antibiotic treatment and may offer insights into the driving factors associated with its recent resurgence.

## 1. Introduction

Group A streptococcus (*Streptococcus pyogenes*; GAS) can cause a wide range of disease manifestations, ranging from uncomplicated superficial infection to severe invasive disease, with a high mortality risk. Scarlet fever represented a serious childhood infectious disease historically. It largely disappeared in developed countries by the mid-twentieth century, coincidental with general improvements in hygiene, sanitation and nutrition and the development of antibiotics. The epidemiological factors associated with declines or rises in the incidence of *S. pyogenes* infection and its clinical manifestation are not well understood, but high mortality rates for GAS have recently been reported [1]. It is important to improve our understanding of the historical dynamics of scarlet fever and the potential driving factors, especially given reports of scarlet fever resurgence in several countries since 2011 [2–6].

Historical data on case notifications over time can convey useful information regarding disease occurrence and seasonal patterns of infection and temporal trends, but to better understand the drivers of infection, estimation of the transmission rate for GAS (causing scarlet fever) via statistical or dynamical modelling, is required*.* Drivers of seasonal variation in *S. pyogenes* transmission and the occurrence of outbreaks can be categorized as ‘biological’, ‘social’ or ‘environmental’. Biological factors concern the health status of the host; poor nutrition might be associated with increased susceptibility to infection. The social aspect is associated with rates of effective contact, such as school attendance or living in overcrowded circumstances, while environmental drivers are associated with survival of the bacteria and effective colonization in the host. Some factors can be classified as both environmental and social, for instance dry versus humid weather conditions might be associated with host colonization and effective contact rates.

Using a historical dataset of scarlet fever notifications in The Netherlands, our main objective was to gain insight into the dynamics of this disease, by investigating the associations of scarlet fever transmission with meteorological and other potential driving factors that might explain temporal variation in cases, and could trigger occasional large seasonal epidemics. Specifically, we explored the extent to which weather factors such as high humidity levels, high temperatures and low seasonal rainfall might facilitate scarlet fever transmission. We also investigated whether the timing of school terms (particularly the beginning of the school year, which occurred in the middle of the summer) was associated with the rate of transmission.

## 2. Methods

### 2.1. Data sources

Weekly data on notified scarlet fever cases from 1906 to 1920 were available at the municipality level from tables appearing in the weekly *Nederlands Tijdschrift voor Geneeskunde*, the main Dutch medical journal. A preliminary step to digitize these reports was required, with extensive data entry error checking undertaken. Case data were stratified into four groups consisting of the three largest municipalities in The Netherlands (during the study period and also at time of writing: Amsterdam, Rotterdam, Den Haag (The Hague)), with the fourth group aggregating together all remaining municipalities. Data were missing for two weeks of our analysis period (i.e. 1908/week 20, 1916/week 38); cases for these weeks were imputed through simple interpolation between the number of cases in the prior and following weeks.

Annual population size (at national and municipality levels) was available from two sources [7,8]. City-level population density in 1900 was also located [9], with density in 1925 calculated from population size. National-level birth rate data were retrieved from Statistics Netherlands [7]. The historical timing of the main school holidays was difficult to locate [10,11]. Around the beginning of the twentieth century schools effectively determined their own summer vacation duration [11]. For each year, we set the summer holiday period at six weeks starting at the beginning of July—with the remainder of the year encoded as term time—recognizing this is an approximation only, as there would certainly have been variation in timing over our analysis period and between cities (electronic supplementary material, figure S1).

The meteorological variables investigated were available at day-level granularity and had been recorded at the centrally located meteorological station in De Bilt and made available online by the Royal Netherlands Meteorological Institute [12]. From these data, we created the following covariates: average weekly temperature, average weekly absolute humidity (in g m^−3^), cumulative winter rainfall (with winter defined as week 49 of previous year through week 9 of index year) and cumulative spring rainfall (over weeks 10–22). (Cumulative summer or autumn rainfall were less relevant as the modelled transmission rates started to rise early in the summer period.) We assumed that temperature and/or humidity would not affect transmission rates in a monotonic (linear) manner, but rather that more extreme weather conditions could be associated with transmission. We, therefore, recoded average weekly temperature and absolute humidity into three categories (low extreme, non-extreme and high extreme), defined as the 1st quintile, quintiles 2–4 and the 5th quintile of the data over the entire study period.

### 2.2. Analysis and modelling approach

Because scarlet fever notifications per time unit do not directly reflect the transmission rate of infection (which is also determined by the proportion of susceptible persons as well as potential other factors), which is the quantity of interest, we derive the time-varying transmission rate from case data through statistical modelling. This required first reconstructing the time-series of susceptibles and then fitting time-series susceptible-infected-recovered (TSIR) models separately for each city.

#### 2.2.1. Reconstruction of the time-series of susceptibles

For an endemic childhood disease setting, susceptible reconstruction methods can be used to estimate the weekly number of susceptible persons. Given data on birth rates and incidence, and ignoring migration, it has been shown that variation in the number of susceptibles around a stable value of the proportion of susceptibles is equivalent to the residuals from the linear regression of cumulative births on cumulative cases [13]. We first describe the number of susceptibles at time *t*, *S_t_*, using the balance equation, equation (2.1). In this equation (applicable to pre-vaccination era childhood infections; referred to as ‘balance’ because the number of births entering the susceptible class is balanced by the number of infected persons leaving), *B_t_* is the number of births at time *t*. Following foundational methodological work [13,14], we estimate the number of true cases of infection at time *t*, *I_t_*, as the number of notified cases divided by the under-reporting factor, *ρ*: *I_t_* = *C_t_*/*ρ_t_*.

2.1 $$S_{t}\;=\;S_{t-1}+B_{t-1}-\;\frac{C_{t}}{\rho_{t}},$$

with

2.2 $$S_{t-1}\;=\;Z_{t-1}+\;\bar{S}.$$

*Z_t_* indicates the residual values obtained after regressing cumulative births on cumulative cases (*Z_t_* indicates the temporal dynamics in the number of susceptibles) while simultaneously estimating the time-varying under-reporting factor (*ρ*) using local Gaussian regression. The mean number of susceptibles over the analysis period, $\bar{S}$, was inferred using equation (2.3) (below) by maximizing the profile likelihood in a generalized linear modelling framework. This step was performed using the *estpars* function of the *tsiR* package for R [14,15].

#### 2.2.2. Estimation of the scarlet fever transmission rate

To estimate the time-varying transmission rate (*β_t_*), we fitted TSIR models to the notified case data separately for the three largest municipalities of The Netherlands. This regression model is specified as follows:

2.3 $$E[I_{t}]\;=\;\beta_{t}\;S_{t-1}\;I_{t-1}^{\alpha }.$$

The TSIR model is a computationally tractable version of the seasonally forced SIR model applicable for diseases with high infectivity, such as for many childhood infections, and relies on the fact that over a long time period the cumulative number of cases is strongly correlated with cumulative number of births. Note that data on notified cases and births enters the model only in equation (2.1). We set the time-step size (*t*) to the approximate length of the generation interval (two weeks) for scarlet fever [16]; the generation interval (the average time between consecutive infection events within a transmission chain) sets the size of the discrete interval [17]. The term *α* is an ‘homogeneity’ parameter and should equal one in the case of mass action. TSIR models are derived by first estimating the under-reporting factor (*ρ*) and the residuals of the susceptible dynamics (*Z_t_*) (see above equation (2.1)) and then rewriting *S_t_*_−1_ in equation (2.3) in terms of the residual susceptibles, and finally converting to the log-linear form [13,14]

2.4 $$\ln\;I_{t}\;=\;\ln\;\beta_{t}\;+\;\ln(Z_{t-1}+\;\bar{S})\;+\;\alpha \;\ln\;I_{t-1}.$$

Posterior estimates for *β_t_* and the ‘homogeneity’ parameter *α* were obtained using Markov-chain Monte Carlo sampling within JAGS [18]. R and JAGS code is available at https://doi.org/10.5281/zenodo.7152996. Two chains of length 30 000 were run, with the first 10 000 samples discarded as burn-in. Convergence and model fit were assessed graphically. For the following univariate analyses, biweekly *β* was converted to weekly *β* via linear interpolation.

#### 2.2.3. Univariate associations between transmission rate and meteorological variables

We calculated the cross-correlations between median estimated transmission rate (using equation (2.4), above) and extreme temperature and extreme absolute humidity, over a range of time lags. These correlations were derived by averaging the cross-correlation values obtained for the three cities. As weather effects on transmission could be immediate or delayed [19], we considered only lags of 0 or more weeks. The lag with the strongest correlation was used in subsequent analyses. We then fitted separate linear regression models for each meteorological variable and each city to visualize the univariate associations with estimated transmission rate. For the relationships for estimated (log-transformed) transmission rate with extreme high/low average weekly temperature, and with extreme high/low average weekly humidity, we plotted the model-predicted log transmission rate for the two extreme quintiles and for quintiles 2–4. Cumulative winter and cumulative spring rainfall were also investigated using univariate models.

#### 2.2.4. Multi-factorial modelling of transmission rate

As the investigated meteorological variables display strong seasonality, it is important to disentangle associations between transmission rate and these variables from associations with school-term timing. Therefore, we adapted equation (2.4) to simultaneously fit terms for the school-term and meteorological covariates

2.5 $$\ln\;I_{t}\;=\;\ln(Z_{t}+\bar{S})+\;\alpha \;\ln\;I_{t-1}+\;\beta_{0}+\beta_{1}T_{t}+\beta_{2}M_{1,t-\mathrm{lag}}+\beta_{3}M_{2,t-\mathrm{lag}}\ldots ,$$

where *β*_0_ is the intercept, *T_t_* is a binary variable encoding school term time (versus main summer holiday) and *β*_2..*k*_ are regression coefficients for the *k* meteorological covariates, *M*_1..*k*,*t*−lag_, (in which extreme temperature and extreme absolute humidity values are from *lag* weeks previously; see above. Note that *β*_2_ and *β*_3_ actually consist of two coefficients to encode the three levels of the corresponding categorical variables). We specified vague Normal priors for all *β* coefficients. Posterior estimates (median and 95% credible intervals) for the regression coefficients, *β_1..k_* were obtained using JAGS.

For estimation of the unique variance explained by the meteorological and school-term time covariates, we fitted multi-factorial additive linear models to the log-transformed transmission rate *β_t_* using ordinary least-squares regression

2.6 $$E[\ln\;\beta_{t}]\;=\;\alpha_{0}+\alpha_{1}T_{t-1}+\alpha_{2}M_{1,t-{\mathrm{lag}}_{1}}+\alpha_{3}M_{2,t-{\mathrm{lag}}_{2}}\ldots .$$

Analogous to equation (2.5), *α*_0_ is the intercept, *T_t_* encodes school term time and *α*_2..k_ are regression coefficients for the meteorological covariates. Unique variance was estimated by removing one covariate at a time and subtracting the *R*^2^ from the *R*^2^ for the full model.

Ordinary least-squares regression analyses were conducted in the R statistical programming environment, v. 3.6.0 [20].

## 3. Results

**Figure 1.**
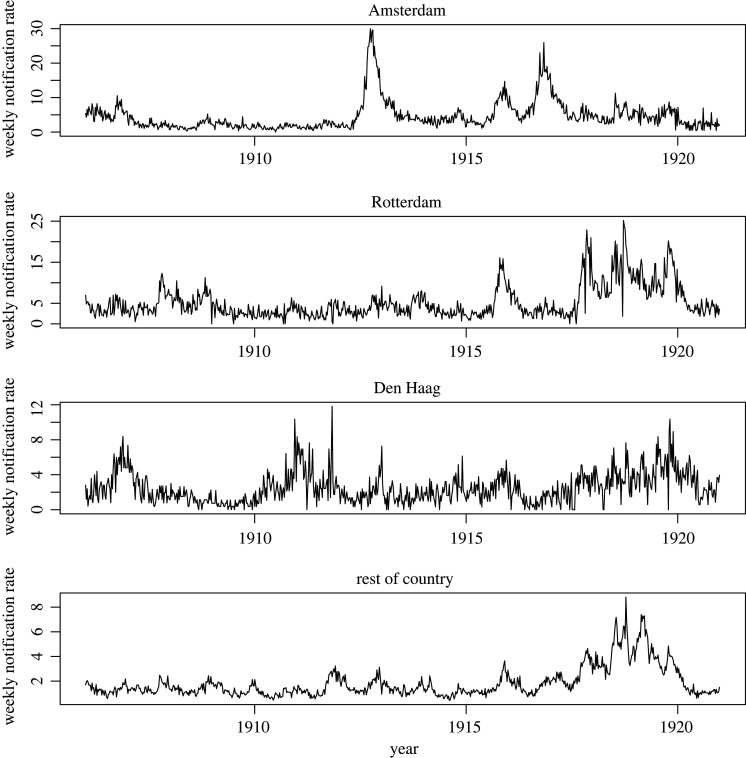
Weekly notification rates (cases per 100 000 population) for scarlet fever (1906–1920), stratified by city (i.e. the three largest municipalities and the rest of The Netherlands).

A total of 116 812 cases of scarlet fever were reported over the period 1906–1920 in The Netherlands. The mean notification rate over the analysis period was highest in Rotterdam, which also had the greatest population density (table 1). Figure 1 shows the case notification rates over the analysis period, stratified by region. There was a prominent rise in notification rates in 1918–1919 present in all time-series except for Amsterdam. Seasonal cycles in notification rates are present, with the most (visually) pronounced seasonal fluctuations apparent only for the three largest municipalities—especially for Amsterdam; these occasional epidemic years did not generally overlap across the three cities. Over all years, the week in which the greatest number of cases were notified occurred most frequently in the last half of September: week 39 (Amsterdam) and week 38 (Rotterdam); for Den Haag this occurred in week 44 (approximately end of October).

**Table 1.** Population size and density in 1900 and 1925, with mean scarlet fever weekly notification rate in 1906–1920, for the three largest municipalities and for the rest of The Netherlands.

City	weekly notification rate (per 100 000 persons)	population (×1000 persons)		population density (persons per km^2^)	
		1900	1925	1900	1925
Amsterdam	4.6	511	712	>3208	4472
Rotterdam	5.2	319	544	3775	6434
Den Haag	2.5	205	391	3313	5915
rest of the country	1.8	4072	5668	—	—
*total*	*3.5*	*5107*	*7315*	*156*	*224*

The process of susceptible reconstruction indicated relatively low variability in *Z_t_* (the estimated number of susceptible persons); the standard deviation of *Z_t_* ranged from 905 (Den Haag) to 1219 (Rotterdam) to 4263 for Amsterdam (electronic supplementary material, table S1; table 1 for city population sizes).

**Figure 2.**
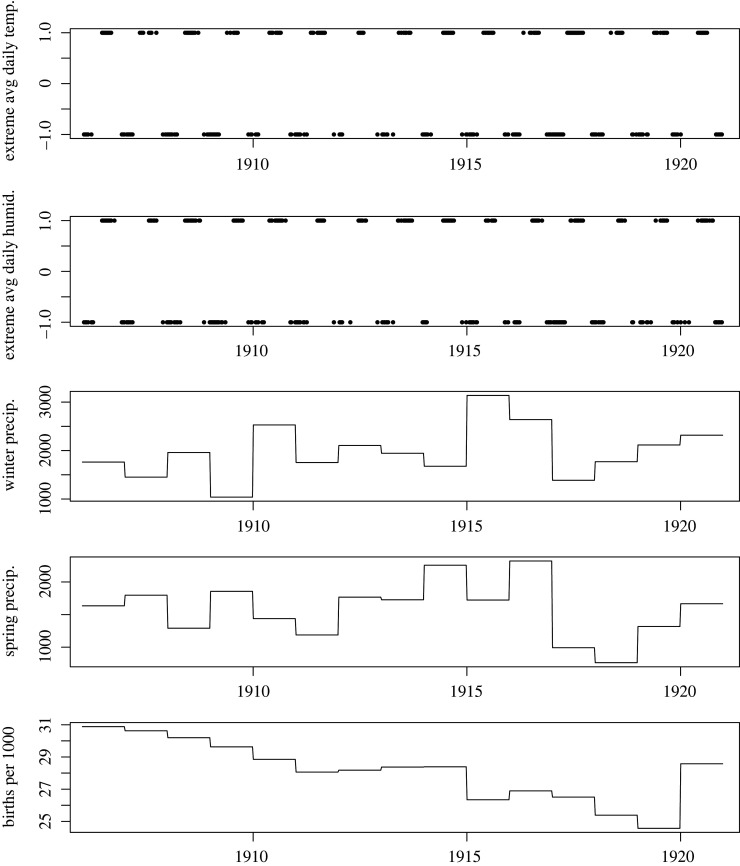
Time-series data for four weather variables and annual birth rate, over the period 1906–1920. (*a*) Extreme quintiles of the weekly average of mean daily temperature (for lowest quintile plotted as −1 and highest quintile as +1); (*b*) extreme quintiles of weekly average of mean daily absolute humidity (plotted on a numerical scale, with the lowest quintile at −1 and highest quintile at +1); (*c*) cumulative winter precipitation (mm); (*d*) cumulative spring precipitation (mm); (*e*) birth rate. All weather variables were recorded at the centrally located meteorological station in De Bilt.

**Figure 3.**
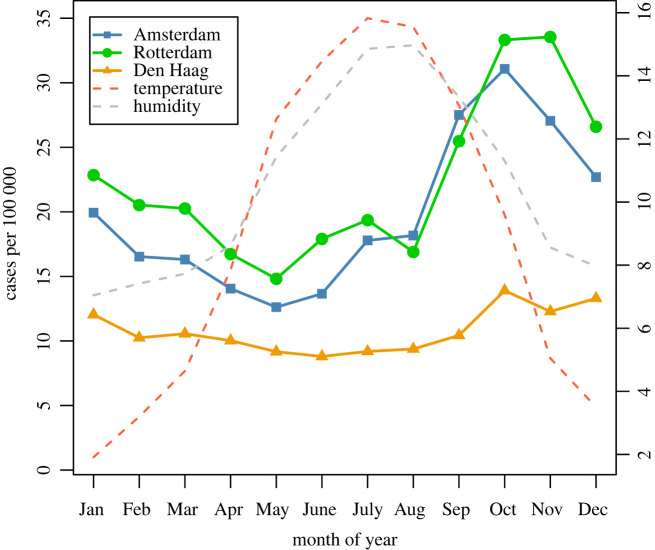
Mean monthly scarlet fever cases per 100 000 (averaged over period 1906–1920), for Amsterdam, Rotterdam and Den Haag, with centrally measured temperature and absolute humidity. The right-hand *y*-axis applies to the two weather variables; for temperature the scale is in degrees Celsius; for humidity the scale refers to absolute humidity * 2 (in g/m^−3^).

**Figure 4.**
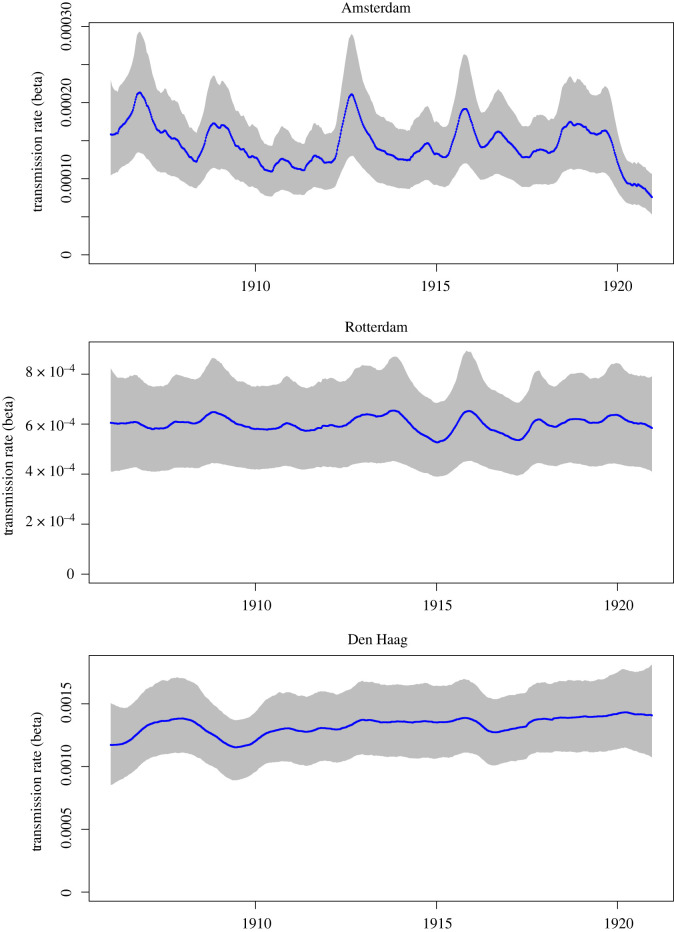
Fitted transmission rate parameter (*β_t_*) over period 1906–1920, for Amsterdam (upper panel), Rotterdam (centre panel) and Den Haag (lower panel). Line indicates the median posterior estimate and shaded region the 95% credible intervals. Note different *y*-axis scales.

**Figure 5.**
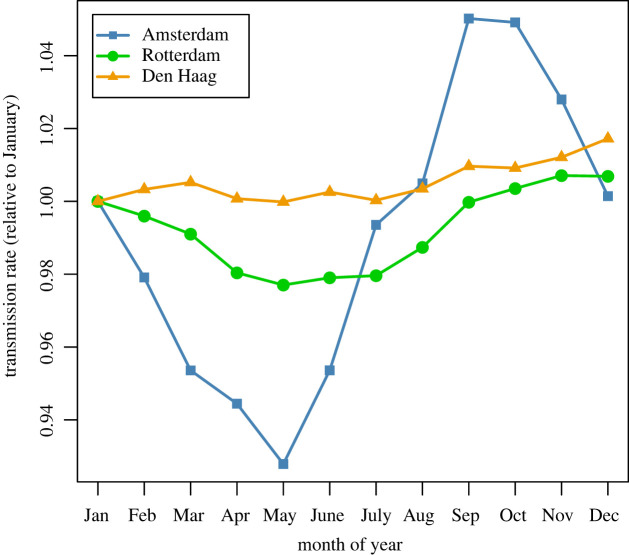
Mean monthly estimated transmission rate, plotted separately for Amsterdam, Rotterdam and Den Haag. Values are relative to January (set to 1.0).

Time-series for the four meteorological variables and birth rate are shown in figure 2. Figure 3 shows the mean weekly number of cases per 100 000 population for each city, averaged over the 15 years of the analysis period, with mean weekly temperature and mean weekly absolute humidity superimposed (also averaged over 15 years). The inferred biweekly transmission rate (*β_t_*) for each city obtained by fitting TSIR models is shown in figure 4, with city-specific seasonality shown in figure 5, and model-fitted parameters are provided in electronic supplementary material, table S1. The highest overall transmission rate (i.e. the number of infections per susceptible person per 14-day period) was estimated for Den Haag (mean *β_t_* of 0.0013 across the entire analysis period), followed by Rotterdam (mean *β_t_* of 0.00060) and Amsterdam (mean *β_t_* of 0.00015).

The strongest correlations between log-transformed transmission rate and extreme high or low temperature and humidity were observed at lags of between 14 and 17 weeks (electronic supplementary material, table S2, figure S3). We used the most recent lag identified (i.e. 14 weeks) to define lagged versions of these variables for use in all subsequent analyses. Univariate regression modelling of log transmission rate (*β_t_*) as a function of each weather covariate indicated positive associations between *β_t_* and extreme high temperature for all three cities (i.e. *β_t_* tended to be higher in periods of the year in which the highest quintile (compared with quintiles 2–4) of temperature was recorded), and positive associations between *β_t_* and extreme humidity (electronic supplementary material, figure S4). There were negative univariate associations between *β_t_* and cumulative winter precipitation for Amsterdam and Rotterdam (i.e. higher winter precipitation was associated with lower transmission in these two cities), and a negative association between *β_t_* and cumulative spring precipitation for Den Haag. Discrepant univariate associations with cumulative winter and cumulative spring precipitation were apparent for Den Haag and for Amsterdam, respectively (i.e. higher winter precipitation associated with higher transmission in Den Haag, and higher spring precipitation associated with higher transmission in Amsterdam).

The unique variance in transmission rate explained by each weather variable varied widely, between 0% and 12.7% depending on city; school term time only explained between 0.05% and 0.56% of the variance (table 2). Collinearity between extreme high/low temperature and extreme high/low humidity was high (correlation coefficients of 0.70 and 0.83, respectively); therefore we retained only the temperature covariate in the multi-factorial analysis. TSIR modelling (equation (2.5)) indicated that adjusting for the other covariates, there was a positive effect of extreme high temperature for all three cities (table 2), and negative effects of extreme low temperature for Rotterdam and Den Haag only. A negative effect of cumulative spring precipitation was observed for Rotterdam and Den Haag, with a positive effect for Amsterdam. For cumulative winter precipitation, there were reliable positive effects for Rotterdam and Den Haag only. There were reliable positive effects of school term time for Rotterdam and Den Haag, but a reliable negative effect for Amsterdam.

**Table 2.** Coefficients (median and 95% credible intervals (CrIs)) from TSIR models including school-term and meteorological covariates, over the period 1906–1920, for the three largest municipalities. Unique variance was estimated from multi-factorial additive linear regression fitted to the estimated (log) scarlet fever transmission rate. Note: coefficients reflect adjustment for other factors in the model.

covariate	Amsterdam		Rotterdam		Den Haag	
	Coef.	(95% CrI)	Coef.	(95% CrI)	Coef.	(95% CrI)
extreme mean weekly temperature (at lag 14 weeks)						
highest quintile	7.0 × 10^−02^	(6.0 × 10^−02^, 8.0 × 10^−02^)	1.8 × 10^−01^	(1.7 × 10^−01^, 1.9 × 10^−01^)	4.1 × 10^−02^	(2.8 × 10^−02^, 5.5 × 10^−02^)
lowest quintile	−1.1 × 10^−02^	(−2.2 × 10^−02^, 1.5 × 10^−03^)	−8.2 × 10^−02^	(−9.6 × 10^−02^, −6.8 × 10^−02^)	−4.0 × 10^−02^	(−5.6 × 10^−02^, −2.6 × 10^−02^)
quintiles 2–4	Ref.		Ref.		Ref.	
*unique variance (R^2^)*	*0.42%*		*0.35%*		*0.21%*	
extreme mean weekly absolute humidity (at lag 14 weeks)						
*unique variance (R^2^)*	*0.25%*		*0.25%*		*0.51%*	
cumul. winter prec.	4.9 × 10^−06^	(−3.1 × 10^−06^, 1.3 × 10^−05^)	3.0 × 10^−05^	(2.1 × 10^−05^, 4.0 × 10^−05^)	7.8 × 10^−05^	(6.7 × 10^−05^, 8.9 × 10^−05^)
*unique variance (R^2^)*	*1.55%*		*0.26%*		*12.7%*	
cumul. spring prec.	1.9 × 10^−05^	(9.3 × 10^−06^, 2.9 × 10^−05^)	−1.9 × 10^−05^	(−3.1 × 10^−05^, −7.1 × 10^−06^)	−6.5 × 10^−05^	(−8.0 × 10^−05^, −5 × 10^−05^)
*unique variance (R^2^)*	*1.05%*		*<0.01%*		*4.63%*	
school term time	−8.7 × 10^−02^	(−1.0 × 10^−01^, −7.5 × 10^−02^)	1.8 × 10^−01^	(1.7 × 10^−01^, 2.0 × 10^−01^)	6.5 × 10^−02^	(4.5 × 10^−02^, 8.4 × 10^−02^)
*unique variance (R^2^)*	*0.05%*		*0.56%*		*0.13%*	

## 4. Discussion

This historical analysis covered 15 years of scarlet fever notifications in three large cities at the beginning of the twentieth century in The Netherlands. We observed multiple pronounced epidemic years in the three cities, but not in the rest of the country, and these epidemic years were not identical in each of the cities. During our analysis period there was a large difference in population density between the three largest municipalities and the rest of the country. It is conceivable that contact rates were higher in large cities compared with the rest of the country, leading to higher transmission rates and therefore to regular and more pronounced epidemics [21].

Scarlet fever transmission may have been additionally influenced by meteorological factors such as relatively high temperatures and low seasonal rainfall, which help shape the seasonal patterns in transmission rate and cases (figures 3 and 5). Our findings are suggestive of a role for extreme high temperature and/or extreme high humidity in the rise in transmission rates beginning in the summer months (figure 5), and for extreme low temperature being predictive of a low transmission rate. The mechanism is unclear due to the long lag time involved; these factors may be correlates of a more generalized seasonal effect. In any case, these factors could only explain a small proportion of the total variance. Total seasonal precipitation might also play a role, as cumulative winter rainfall uniquely explained a relatively high proportion of variance for Den Haag (12.7%), and a dry spring was associated with an increased transmission rate for Rotterdam and Den Haag, consistent with analyses of historical scarlet fever mortality data from England and Wales [22]. Again, the mechanism is unknown but could relate to host factors (susceptibility). However, the univariate associations for both rainfall variables, and the adjusted associations for cumulative spring rainfall, were inconsistent in direction between cities. Variability in the estimated number of susceptibles in the population was relatively small, thus suggesting that depletion of the pool of susceptibles was probably not a strong determinant of across-season variation in scarlet fever incidence, in contrast to what has been observed through modelling infections such as measles [13].

As also observed within a similar time period (1907–1931) in Copenhagen [17], several epidemic years in The Netherlands occurred in the early 1900s, even though scarlet fever in The Netherlands was declining before this time (based on mortality data; [23]), and notified case rates declined again from about 1920. Our work complements the Danish evidence, by showing that epidemic years were only apparent for the three largest cities, and the existence of different patterns in estimated transmission rate between cities of reasonably similar size (but differing in population density) located only 50–100 km apart.

Our results are partly consistent with findings of studies investigating associations between meteorological variables and contemporary scarlet fever outbreaks. Lu *et al*. [24] demonstrated positive lagged correlations between scarlet fever incidence with weekly temperature range and atmospheric pressure in Guangzhou City, China, as well as a negative correlation with weekly aggregate rainfall. In a second Chinese study, Zhang *et al.* [25] reported positive associations between scarlet fever incidence and temperature in Jiangsu province. The mechanisms by which meteorological factors could impact the transmission of bacterial infection are not well understood. One hypothesis could be that hot weather affects the host by creating favourable conditions for nasopharyngeal colonization, which then leads to a boost in disease cases. However, such a hypothesis is not supported by the seasonality of contemporary scarlet fever, for which peak incidence occurs in late winter or early spring in temperate climates [26,27].

Multi-factorial TSIR analysis indicated that the meteorological factors were independent of the ‘social’ driver investigated, school term time, which after controlling for the other covariates was positively associated with scarlet fever transmission in Rotterdam and Den Haag. School term/holiday timing—a proxy for varying contact rates between schoolchildren—may be a partial driver (see electronic supplementary material, figure S1), but its role is less clear than for other infections, as shown by Metcalf *et al.* [17] using a historical Copenhagen dataset and a TSIR modelling approach. The authors observed a strong increase in scarlet fever transmission during late summer, before the end of the school summer vacation, which was also observed for diphtheria and varicella), and concluded that term-time forcing cannot explain this increase. Our data partially concur; for some years, particularly visible in the data for Amsterdam (electronic supplementary material, figure S1), the notification rate began to rise during the school summer holiday yielding a peak in incidence within term time. In The Netherlands, school (including home schooling) was compulsory for children aged 6–12 years only starting in 1901 [28]. The group of non-school attenders in this age group and children younger than 6 or older than 12 years may have been large enough to sustain transmission after the start of the term [29], which would predict only a modest degree of term-time forcing.

A resurgence of scarlet fever, beginning around 2011, has been reported by a number of countries. For instance, increased case notification rates have been observed since 2013/14 in England [2,3], since 2011 in Hong Kong [4] and in mainland China [5], and since 2013 in South Korea [6]. Of particular concern was the recent (in 2016) increase in scarlet fever and invasive GAS disease in England [3,30] leading to severe complications and hospitalization.

Several possible drivers for the return of scarlet fever have been proposed (see also [31,32]), depending on the region; these include emergence of new strains or transmission of known genetic element by horizontal gene transfer, for which population immunity is lacking [27,33,34], social factors such as overcrowding in classrooms and hospitals; rising birth rates (leading to increased number of susceptibles), and concurrent circulation of other ‘pre-disposing’ pathogens, such as respiratory viruses [33]. One prominent finding—emergence of a new *emm1* lineage—offers a plausible explanation for the rise of invasive disease in England in 2016, but not of the initial increase in cases in 2013/14 [27]. Genetic mutations and antimicrobial resistance have also been put forward as factors for Hong Kong and Chinese settings [34]. In analyses of historical data, weather factors such as low absolute humidity [35] or a dry versus wet spring/summer season [22] have been proposed, and social factors affecting contact rates (e.g. school holiday timing) [17] have been tested. The observed resurgence in scarlet fever is largely based on surveillance (case) data; the present historical analysis suggests that interpretation of the recent outbreaks may benefit from estimation of transmission rates.

Although our model-based analysis has a number of strengths, we note several limitations*.* First, we used available historical data on weather factors measured at a central location in The Netherlands in all analyses; however, there is probably little meaningful variation in these factors between the three largest municipalities, which are located only 50–100 km from each other. Unmeasured confounders, such as the density of occupants per household, may have contributed to the observed between-city variation in effect directions for some weather variables. Information on age at case notification was not available; we assumed that cases occurred mainly among school-age children; this was deemed reasonable as the majority of historical scarlet fever cases in Copenhagen were observed between 5 and 15 years of age [17]. Furthermore, the fitted case under-reporting parameters may capture factors other than completeness of case reporting.

We lacked data regarding other potential driving factors affecting scarlet fever transmission. Food shortages/nutritional deficiencies during the last years of WWI—bread rationing had begun in 1917—might account for part of the high incidence observed around that time, if poor nutrition increased susceptibility to infection [36,37]. The 1918/19 influenza pandemic occurred near the end of our analysis period, and we note that notification rates were relatively high in these years in all regions except Amsterdam. Given that complications of GAS infection may be more severe when secondary to influenza [38], such an interaction could potentially explain these high notification rates. Finally, we did not attempt to include the partial school closures that were in force at this time [39], but these took place after the modelled rise in transmission rate.

## 5. Conclusion

This historical analysis of scarlet fever notifications in The Netherlands in the early twentieth century has shown associations between seasonal variables—namely meteorological factors (hot temperatures, high seasonal precipitation levels)—and increases in scarlet fever transmission, which both accounted for late-summer peaks in notified cases and may have contributed to the occurrence of occasional epidemic years. However, the roles of these factors were limited and not consistent over the three cities. The fact that pronounced epidemics did not occur in the same year for all cities cannot be explained by weather factors alone—and in our analysis between-city variation is unlikely to be accounted for by such factors, even if city-specific meteorological data were available.

The recent rise in scarlet fever incidence reported in several countries could be partly due to changing climate conditions. Similar analyses to ours could be applied to better understand the recent incidence trend in England. For instance, three recent years (2014, 2017, 2018) were among the 10 hottest years on record in the UK [40]; weather conditions may have contributed to increasing transmission over a low endemic level of *S. pyogenes*. However, the data from China may be more challenging to explain by local variability in weather factors, as a rise in scarlet fever incidence was observed across most regions. Nevertheless, moderate changes in meteorological factors—as we inferred for historical data in The Netherlands—should not be ruled out as part explanation for the return of scarlet fever to England and China.

## Data Availability

Weekly scarlet fever case data for our analysis period are supplied as a supplementary file. R and JAGS code has been deposited at the publicly available repository zenodo (https://doi.org/10.5281/zenodo.7152996). The data are provided in electronic supplementary material [41].
